# Nanoscale Footprints of Self-Running Gallium Droplets on GaAs Surface

**DOI:** 10.1371/journal.pone.0020765

**Published:** 2011-06-06

**Authors:** Jiang Wu, Zhiming M. Wang, Alvason Z. Li, Mourad Benamara, Shibin Li, Gregory J. Salamo

**Affiliations:** 1 Arkansas Institute for Nanoscale Material Science and Engineering, University of Arkansas, Fayetteville, Arkansas, United States of America; 2 State Key Laboratory of Electronic Thin Films and Integrated Devices, University of Electronic Science and Technology of China, Chengdu, People's Republic of China; Queen's University at Kingston, Canada

## Abstract

In this work, the nanoscale footprints of self-driven liquid gallium droplet movement on a GaAs (001) surface will be presented and analyzed. The nanoscale footprints of a primary droplet trail and ordered secondary droplets along primary droplet trails are observed on the GaAs surface. A well ordered nanoterrace from the trail is left behind by a running droplet. In addition, collision events between two running droplets are investigated. The exposed fresh surface after a collision demonstrates a superior evaporation property. Based on the observation of droplet evolution at different stages as well as nanoscale footprints, a schematic diagram of droplet evolution is outlined in an attempt to understand the phenomenon of stick-slip droplet motion on the GaAs surface. The present study adds another piece of work to obtain the physical picture of a stick-slip self-driven mechanism in nanoscale, bridging nano and micro systems.

## Introduction

Droplets have received increasing attention for potential applications in lab-on-a-chip, droplet epitaxy, and micro/nanofluids.[1]–[6] Gallium (Ga) droplets are currently of great interest for their potential applications in advanced quantum devices; these droplets can be transformed under irradiation of group-V molecular beams into various semiconducting nanostructures such as quantum dots and rings.[7]–[17] Recently, Tersoff *et al.* and Hilner *et al.* found a new type of self-driven motion of Ga droplets on the III-V crystalline semiconductor surfaces through the thermal decomposition of semiconductors.[1], [2] The movement of the droplets was investigated by using mirror electron microscopy (MEM) in real time. In terms of dynamic events, MEM is a very useful *in situ* tool. Unfortunately, it does not provide a direct observation of the nanoscale structures induced by the droplets due to limited resolution.[2] To further study how microscale droplets depend on nanoscale surfaces, Hilner *et al.* applied low energy electron microscopy and scanning tunneling microscopy to explore self-propelled Ga droplets on a GaP(111)B surface.[2] Their results show dependencies of motion on the nanoscale ordering of surface step structures. These demonstrations open up new opportunities for novel nanofabrication. However, a detailed picture of the nanoscale structures resulting from the Ga droplet movement on the GaAs surface is lacking. Further investigation is needed to provide deeper insight into the self-driven motion during the Ga droplet moving process.

Detailed footprints of self-driven droplet motion (spontaneous droplet motion or self-propelled motion) are investigated in this article. Using scanning electron microscopy (SEM) and atomic force microscopy (AFM), footprints from the primary running droplet are reviewed in greater detail, showing nanoscale terraces as a result of stick-slip motionand findings on the nanoscale footprints of Ga droplets on a GaAs surface are presented in this study. For example, after the collision of two droplets, an atomic flat surface is exposed and secondary droplets are ordered along the boundary between the newly exposed surface and surrounding area. Further investigation including various techniques[1], [2], [18], [19] is needed to provide a deeper insight of into the self-driven motion during the Ga droplet moving process.

## Results and Discussion

**Figure 1:**
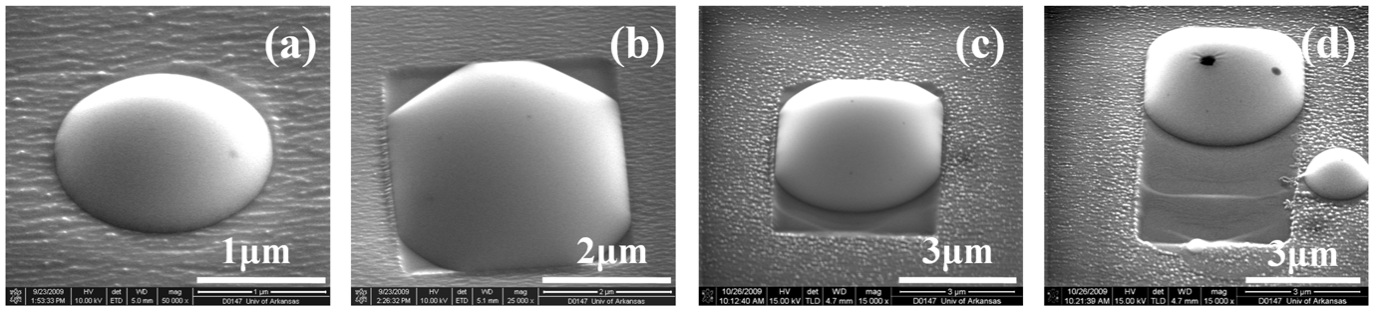
The evolution of self-driven droplets. The development process from immobile droplet to self-driven droplet. SEM images in a 45-degree viewpoint are as follows: (a) a “baby” stage droplet with circular contact line; (b) a “child” stage droplet with rounded-rectangular shape contact line, is learning to walk; (c) is a “teenage” droplet with hexagonal-face shape contact line, is in the beginning stage of walking; and (d) is an “adult” droplet with a hexagonal-face-shape contact line, is in the early stage of stick-slip motion.

In this study, the sample is prepared in a molecular beam epitaxy (MBE) chamber. Upon removal from the growth chamber, a large milky area can be seen on the surface of the sample with unassisted naked eyes. This milky region is, in general, comprised of Ga droplets which are the result of a non-congruent evaporation of GaAs at high temperatures.[20] With high resolution SEM and AFM in tapping mode, Ga droplets and their trails are observed clearly indicating droplet motion. It is worthwhile to mention that the energy dispersive X-ray spectroscopy (EDS) has confirmed that these droplets are pure metallic gallium. Figure 1 shows evidences of Ga droplet motion in the ±[110] directions and the evolution patterns are observed. The confinement of motion along [110] can easily be understood as the effects of surface anisotropy, however, the evolution of running droplets appears mysterious. Although a thermodynamic model on the driving force of running droplet has been proposed, the question of how running droplets interact with the crystals on the underside remains unsolved. As displayed in Figure 1 (a), the droplet first starts with a round shape. As the droplet continues to increase in size, it melts down the underside of the crystal and its circular interfacial area changes into a rectangular interfacial area, as shown in Figure 1 (b). The rectangular boundary suggests that the mature droplet forms its solid-liquid interface well below the substrate surface, so that crystal anisotropy takes effect to confine its contact line along ±[110] and ±[1]–[10] directions on GaAs (001) surface. Finally, the droplet breaks out and moves along [110] direction, as shown in Figures 1 (c) and (d). The droplets are in running status with a contact line in a hexagonal-faced shape. The back side of the leading droplet noticeably becomes a circular arc, which indicates that it is relaxed and without constraint. These observed images will have important consequences for modeling droplet behavior.

**Figure 2:**
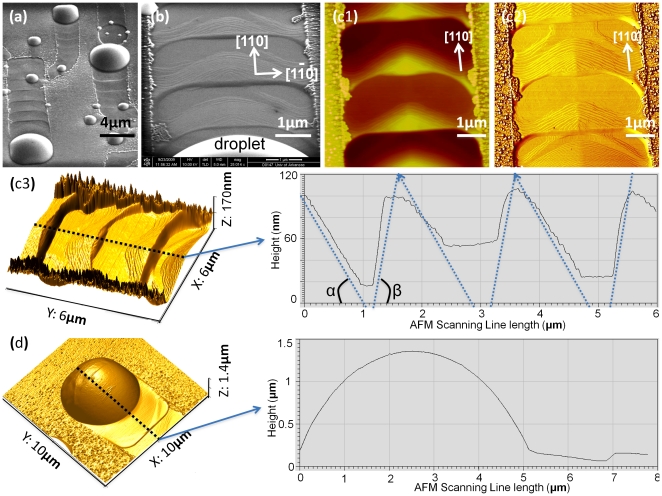
The morphology of running Ga droplets. The typical footprints of Ga droplets motion are: (a) a SEM image of running droplets in microscale; (b) a SEM image of the detailed footprints left behind by the leading droplet in nanoscale; (c) AFM images of the footprint from droplet motion: (c1) the simple topographical AFM mapping; (c2) AFM phase image highlighting the stepping nanostructures of footprints; and (c3) AFM 3D images revealing the depth of the footprints with the central dashed-line corresponding to the AFM line-scanning profile on the right-hand side graph. The multiple dashed-lines on the scanning-line graph indicate the footprint are featured in angles α and β (α = 1.6° and β = 6.4° after ratio normalization). Finally, (d) is the AFM 3D image of a moving droplet, with the central dashed-line corresponding to the AFM line-scanning profile on the right side.

Previous studies have shown that the force on droplet is proportional to its size. The droplet has to be large enough for the thermodynamic force to break the droplet out evidenced by droplet evolution. The footprints left by the running droplets also contain important information on the correlation between droplet motion and footprints. In order to gain further insight into the self-propulsion process, the historical steps caused by the process are investigated as shown in Figure 2. Particularly, the well-ordered nanoscale footprints left behind by the leading droplet are observed in detail as shown in Figures 2 (a), (b) and (c). The depth of each footprint is in the range of 100 nm as indicated in Fig.1 (c3). Figures 1 (b) and (c) show that the highly ordered surface left by the leading droplet consists of periodical footprints; each period is made up of approximately 30 steps, the height of each surface step being ∼3 nm, and the lateral displacement between adjacent surface steps being ∼30 nm. These well-ordered footprints clearly suggest a stick-slip motion of droplets as well as periodic interaction between droplets and the crystal on the bottom of the droplets. Such interaction is believed to be local etching of the GaAs surface by Ga droplets.[11], [21]–[23] The AFM measurements show featured angles (α≈1.6° and β≈6.4°) derived from the periodic nano-terrace footprints from the stick-slip motion of primary droplet, as plotted on the AFM scanning-line profile in Fig. 2 (c3), which are related to certain GaAs crystal facets. The self-propelled droplet is about a few microns in diameter, which is reasonably similar to the values observed by Tersoff *et al* and Hilner *et al*.[1], [2] The height of a droplet is about 1.4 µm as shown in Figure 2 (d), which is different from the relatively flat Ga droplets observed by Hilner and coworkers on GaP (111) substrates. Additionally, the ordered steps are not perpendicular to the droplet trails but have an approximate angle of 60° off the trails. The angled steps indicate the moving droplets have ordered the bottom surface along certain crystal orientations to minimize the system energy.

**Figure 3:**
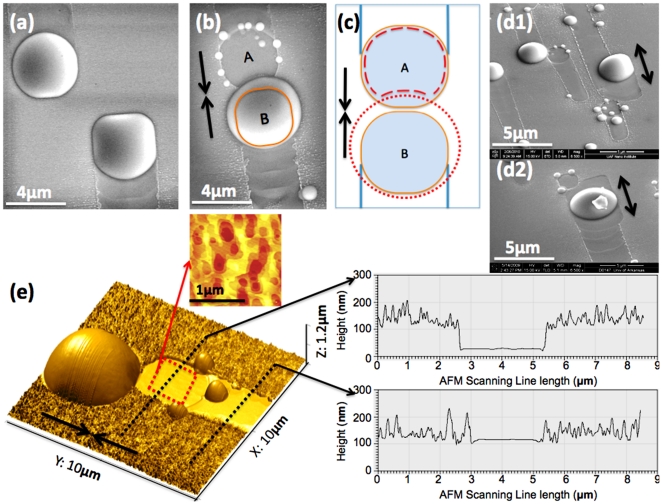
Collision between two running droplets. The two droplets are coincident with each other: (a) Before colliding, in top view of SEM, both the shape of droplets A and B are confined in their moving trails. (b) After colliding, in the top view, droplet A merges with droplet B. The orange circular shape indicates the original droplet B before colliding resulting in an empty well with a group of small droplets in position A. (c) the top view shows the schematic diagram of the droplet coalescence. The orange circle indicates the droplet before collision; the red dot-dashed circle indicates the coalescent droplet, and the red dash line in position A indicates the roundish well after collision. Both (d1) and (d2) are SEM images which show that the resultant larger droplets from coalescence are moving backward and forward. (e) This shows the AFM 3D image of the coalescent event. The red dash-square area corresponds to the simple topographical AFM mapping (top side) in which the atomic scale layers highlight the atomically flat bottom of the well. The two black dash-lines correspond with the AFM line-scanning profile graphs (right side), outlining the significant depth of the well by comparing them with the trail.

Another fascinating observation of the system comes at the collision of two droplets. Here, we find the formation of a relatively deep, roundish well after the coalescence of two droplets, as shown in Figure 3. Two droplets moving toward each other are also shown in Figure 3 (a). As illustrated in Figure 3 (b), a big droplet appears larger than the original droplets at both the A and B positions prior to a collision at its edge outside of the original boundary provided by its trail. In dramatic opposition, position A, which is relatively deep in comparison to the trenches found from the motion of droplets, has been occupied by a droplet that has merged into the droplet in position B. Here it is evident that two droplets which were in position A and B do collide with each other, and merge to form one larger droplet in position B. There are also several tiny droplets sitting around spot A with sizes that are less than 500 nm in diameter.

**Figure 4:**
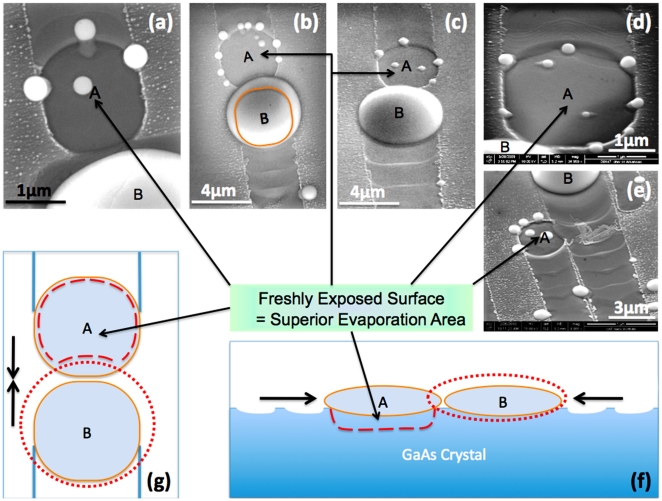
Freshly exposed surfaces from colliding events. Images (a), (b), and (c) are SEM images of coincident events in top view. Both (d) and (e) are SEM images of coincident events in a 45-degree viewpoint, where roundish deep wells are clearly visible in the A positions of each. Both (f) and (g) are schematic diagrams of the droplet coalescence. (e) In the top view, the orange circle indicates the droplet before the collision; the red dot-dash circle indicates the coalescent droplet, the red dash line in position A indicates the roundish well after collision. (f) In the cross-section view, the orange ellipse indicates the droplet before colliding; the red dot-dash ellipse indicates the coalescent droplet, and the red dash line in position A indicates the roundish well after collision. The wavelike surface indicates the footprints left behind by the droplets.

Based on the fact that significantly deeper wells are formed right after these collision events take place, as shown in Figures 3 (b), (d), (e), and Figures 4 (a)–(g), it suggests that the suddenly exposed fresh-surface is equivalent to a superior evaporation area.[24] Also, the solid-liquid interface between droplet and substrate is an unstable layer with dynamic processes.[25], [26] A sudden exposure of this nonequilibrium interface causes a superior evaporation process, so thatthe resultant evaporated well is much deeper and flatter than the initial interface beneath the droplet. Noticeably, as measured by the topographical AFM mapping in Figure 4 (e), the bottom of the well is nearly atomically flat, its flattening ratio as high as 347∶1 (width range of 1.7 µm: height range of 4.9 nm). The depth and pattern of these roundish wells are reasonably similar to those depressions from the chemically selective removal of Ga clusters on GaAs surface, which indicates interaction between droplets and substrates during droplet motion.[27] There is a group of small droplets sitting on the surrounding ridges of freshly exposed areas, as shown in Figures 3 (b), (d), (e) and Figures 4 (a)–(e). These small droplets come from the evaporation process locally in stead of the remaining liquid Ga from the leading primary droplet in the A position.[24] Thus, the appearance of a group of small droplets does support this superior evaporation argument which will have crucial consequence for the analysis of droplet motion. For example, the moving direction of a droplet can be either +[110] or –[110] and once it moves towards one direction accidently, such as a cause of thermal fluctuation. It is favoring to keep going in the same direction, however, until certain sudden change of the surface dynamics, such as a collision of two droplets, the droplet can reverse the direction in which it is moving. As shown in Figures 3 (d1) and (d2), we captured the merged droplet moving backward even though the front side is a “rougher” surface. The phenomena suggest different surface energies between the ordered trail area and the newly exposed surface.

The driven forces of the droplet motion are complicated due to involvement of surface dynamics, surface morphology, and droplet-substrate interaction. However, the motion of the droplets can be accounted for several reasons : (i) there is a rough surface in the front of the leading droplet which will create roughness-related force to drag the droplet;[2] (ii) the difference between vapor-solid and liquid-solid interface energies across the tri-junction;[1] and (iii) the gradient in free energy underneath the droplets.[28], [29] Based on the observation of the nanoscale footprints of running droplets and droplet collision events, we are able to sketch a picture of droplet motion in order to gain a deeper insight into the phenomenon of self-driven droplets: A small droplet gains Ga through absorbing Ga from the surrounding surface diffusion and underside substrate melt, which increases its volume. While the droplet is building up its volume and melting down its underside, its circular interfacial area is changing into a rectangular interfacial area. This corresponds to the “stick” state of the stick-slip motion. The forces caused by surface energy difference continue to increase. When the surface tension of the droplet reaches the threshold of binding its cohesive body, random perturbation instantly triggers the droplet's surface breakout, therefore, releasing Ga liquid to flow out, and proceeding a distance approximately 1/3 the size of a droplet. This transient change corresponds to the “slip” state of the observed stick-slip motion. As the droplet moves forward, it suggests that the propulsion mechanism proposed by Hilner et al. plays a role in this “uphill” state,[2], [30] where the roughness of the gallium “lava” covered area combined with interfacial free energy difference is able to steer droplet motion and order the surface beneath the droplet. The distance moved during the “slip” state is related to the surface friction of the newly buried rough surface. The increasing surface friction brings the running droplet back into the stick state. After ordering the underside crystal, the droplet friction decreases and the droplet returns to the slip state again. Keeping the above “stick” and “slip” process going, the resulting footprints fit with the experimental fact.

## Materials and Methods

### Sample preparation

A semi-insulating epitaxial ready GaAs(001) wafer was prepared in the ultra high vacuum at 350°C over 1 hour. Sequentially, the wafer was transferred into a MBE chamber and deoxidized thermally at 600°C for ten minutes. The oxide desorption process was monitored by reflection high-energy electron diffraction (RHEED) *in situ*. After thermal oxide desorption, a 500 nm thick GaAs buffer layer was epitaxially grown at 1 ML/s at 580°C. Then all the source cells were closed and after the background pressure reach below 10^−8^ Torr, the sample was heated up immediately to 680°C with a ramp rate of 50°C/minute. The high temperature was maintained until dramatic increase of background pressure (∼10^−6^ Torr) due to GaAs decomposition. The sample was kept rotating through the entire process. Subsequently, by switching off the power supply of heater, the sample was cooled down to room temperature immediately.

### Characterization

The morphology of the sample was characterized by a FEI Nova NanoSEM and Veeco AFM, respectively.
